# A Review of Apps for Calming, Relaxation, and Mindfulness Interventions for Pediatric Palliative Care Patients

**DOI:** 10.3390/children5020016

**Published:** 2018-01-26

**Authors:** Taelyr Weekly, Nicole Walker, Jill Beck, Sean Akers, Meaghann Weaver

**Affiliations:** 1 Department of Cardiology, University of Nebraska Medical Center, South 42nd Street and Emile Street, Omaha, NE 68198, USA; tjmiller@unmc.edu; 2 Children’s Hospital and Medical Center 8200 Dodge Street, Omaha, NE 68114, USA; niwalker@childrensomaha.org (N.W.); jibeck@childrensomaha.org (J.B.); sakers@childrensomaha.org (S.A.)

**Keywords:** technology, mobile applications, meditation, multimedia, children, palliative, relaxation, stress

## Abstract

Patients and families increasingly use mobile apps as a relaxation and distraction intervention for children with complex, chronic medical conditions in the waiting room setting or during inpatient hospitalizations; and yet, there is limited data on app quality assessment or review of these apps for level of engagement, functionality, aesthetics, or applicability for palliative pediatric patients. The pediatric palliative care study team searched smartphone application platforms for apps relevant to calming, relaxation, and mindfulness for pediatric and adolescent patients. Apps were reviewed using a systematic data extraction tool. Validated Mobile Application Rating Scale (MARS) scores were determined by two blinded reviewers. Apps were then characterized by infant, child, adolescent, and adult caregiver group categories. Reviewer discussion resulted in consensus. Sixteen of the 22 apps identified were included in the final analysis. The apps operated on either iOS or Android platforms. All were available in English with four available in Spanish. Apps featured a relaxation approach (12/16), soothing images (8/16), and breathing techniques (8/16). Mood and sleep patterns were the main symptoms targeted by apps. Provision of mobile apps resource summary has the potential to foster pediatric palliative care providers’ knowledge of app functionality and applicability as part of ongoing patient care.

## 1. Introduction

Technology represents an entertainment presence for culture; this anecdotally is recognized in the increased presence of audiovisual material use as a distraction technique by parents in waiting rooms and hospital rooms. Approximately three-quarters (77%) of Americans now own a smartphone and half own a tablet computer [1]. Smartphones are near universal among younger adults, with >90% of adolescent and young adults owning a smartphone [1]. With the widespread ownership and access to technology, our pediatric palliative care team wondered whether we may consider leveraging current technology use to include apps for calming, relaxation, and mindfulness rather than strictly gaming apps. 

In considering the role of technology in a pediatric clinical setting, our study team considered the roles of both relaxation and distraction (Table 1). Our study team differentiated between relaxation and distraction in terms of the level of participant engagement/activity required. A relaxation app was one that involved active entrance into a focused state of calm such as participatory mindfulness, engaged visualization, or body scan. A distraction app was defined by the study team as one that included a passive receptivity to sound/visual diversion or recreation. Both relaxation and distraction apps each shared an endpoint of a lifting of tension, soothing of anxiety, and restoration to a sense of peace. The vibrant imaginations and full engagement of pediatric patients means that even a distraction app can foster a tranced-equilibrium of deep relaxation. 

Electronic interventions such as mindfulness apps and relaxation-based apps have been noted for their positive effect on the general health and psychological well-being of patients with chronic, complex medical conditions [2,3,4]. Technology distraction is noted to be accepted by pediatric patients [5,6] with improvement in pain management and cooperation.

Nervous or anxious feelings are noted as a significant symptom for children in medical settings [5]. The aim of our study was to investigate what apps for relaxation and distraction exist for pediatric palliative patients and to describe the features, qualities, and intended audience.

## 2. Materials and Methods

### 2.1. Eliciting Pediatric Relaxation Apps

Smartphone application platform stores, Blackberry World App (Blackberry, Waterloo, ON, Canada), App Store iOS (Apple Inc., Cupertino, CA, USA), and Google Play (Google, Mountain View, CA, USA), were searched between May and July 2017 using keywords: child, pediatric, adolescent, palliative, mindfulness, relaxation, and calm. An announcement was posted on a national Child Life e-mail list to gather additional relaxation application recommendations.

### 2.2. Procedure for Reviewing the Relaxation Apps

Three reviewers (MW, NW, AW) independently performed eligibility assessments of apps utilizing a pre-determined eligibility checklist. Inclusion criteria included pediatric-specific apps, free apps, and privacy-protecting apps. Exclusion criteria included apps which were not pediatric-specific, apps with a cost, and any app with “open discussion” format of interaction electronically (to protect children from exposure to unknown co-app users). These independent reviewers reached consensus for exclusion/inclusion decision with >88% inter-rater agreement. Five apps were discussed to reach inclusion/exclusion consensus.

The team developed a data extraction sheet in Microsoft Excel (Microsoft, Albuquerque, NM, USA), which underwent a pilot test on three randomly selected apps. Items on the extraction sheet included app name, cost, platform availability, brief summary of the app as posted by the app store, listing of app features, targeted age group, and consumer rating. Each app was reviewed for specific mention of symptom profiles such as stress, anxiety, bullying, trauma, sleep disorders, and depression. Each app was further reviewed for specific mention of relaxation approaches such as stress management, symptom tracking, body scanning, calming audio, diary or journaling, meditation, mood tracking, hypnosis, cognitive behavioral therapy, crisis management, yoga or body movements, brainwave frequencies, spirituality/religious support, breathing techniques, or heart rate and breathing tracking. The data extraction sheet included items for study team members to indicate their perspective on the positive and concerning features of each app, age appropriateness, and level of app interaction (specifically whether the app could be used for children with fine motor skill limitations). Reviewers provided descriptions of app relevance to pediatric palliative care. 

Reviewers utilized the Mobile Application Rating Scale (MARS) classification score to measure app aesthetics, engagement, functionality, and overall quality [7]. The MARS is a validated, objective, and reliable tool for assessing the quality of mobile health apps [7]. The 23-item MARS questionnaire results in a mean score from 1–5 specific to engagement, functionality, aesthetics, and information domains. Higher mean score per domain represents higher quality.

A team of six reviewers (MW, AW, SA, JB, NW, TM) from disciplines of palliative care, psychology, child life (child development specialist), and nursing systematically reviewed apps in full with two reviewers downloading and using each app program a minimum of one session per reviewer. These two study team members independently completed the data extraction per app and each reviewer entered the data into an Excel extraction template designed by two study team members (MW and AW) to enable consistent data formatting for team analysis. A minimum of one additional study team member also downloaded each app, utilized the app for a full session, and checked data extraction to recognize differences of opinion and recirculate these findings back to primary and secondary reviewers for agreement.

### 2.3. Data Analysis

Data analysis followed a pre-determined quantification of extracted items and content analysis. This approach facilitated the recognition of patterns, variations, and relationships from extracted data. The two reviewers’ MARS scores were averaged per engagement, functionality, aesthetics, and information domains.

## 3. Results

A total of 22 apps were identified with two then excluded on full application review as they were not pediatric-specific, three excluded due to cost, and one excluded due to “open discussion” format of electronic interaction. Two nonduplicative applications were added from the listserv project announcement. This resulted in 16 total apps for inclusion. 

Apps used either the iOS (Apple Inc.) or Android (Google) operating systems with 15/16 (94%) available on both platforms. All of the apps included were available in English, with four of the apps available in Spanish. All of the included apps were available for free. Nine of the apps were identified as potentially relevant for the infant group, 14 for the elementary group, 16 for the adolescent group, and 15 for the adult caregiver category. The study team analyzed the apps for level of required interaction to determine each app’s feasibility for children with limited fine motor coordination, of which eight of the 16 (50%) apps could be started and then required no further user-initiated action.

Symptoms specifically targeted by the apps included: stress (50%), sleep disorders (38%), anxiety (38%), general mental health (25%), and depression (19%). There were two apps that did not fit any of the specifically mentioned symptoms (Fluid app [8] and Kindoma Drawtime app [9]).

A majority of the apps, 12/16 (75%), featured a relaxation approach and 11/16 (69%) used a stress management technique. A total of 8/16 (50%) featured soothing images and 8/16 (50%) included a guided breathing technique. Apps targeted specific coping mechanisms such as: calming music (44%), calming words (44%), mindfulness approach (38%), meditation (38%), yoga (31%), body scan (25%), symptom tracking (19%), mood tracking (19%), cognitive behavioral therapy (13%), games (13%), and brainwave frequencies (6%). 

MARS domain scores are available in Table 2.

## 4. Discussion

Increased availability and acceptance of mobile technology offers interesting, creative, and vibrant exposure to various relaxation techniques. However, this requires provider knowledge of what apps exist and what activity options are available. The study team utilized a novel systematic approach to investigate apps by assigning two blinded reviewers per app and by utilizing a validated app quality rating tool (MARS). This approach to app review was entertaining and educational for reviewers but was also challenging due to the seemingly subjective nature of app approval (interestingly, inter-rater agreement was high at 88%). This study offers the foundational information needed to inform providers of available applications which can serve as the base for increased use of technology for calming, relaxation, and mindfulness.

While our study team prioritizes the role for human interaction in calming techniques and strategies, the reality is that many of our patients currently already use “gaming” as a form of distraction. As a care team, we have collectively opted to still prioritize and emphasize relational relaxation techniques as our primary relaxation intervention. For those families who are already incorporating technology for distraction, we pursued app review to emphasize the relaxation component of technology use rather than just the distraction component. Our study team is now working on creating a handout of calming, relaxation, and mindfulness app resources for patients and families and loading tablets with symptom-specific apps for children and families to “try out” with a knowledgeable care provider while in the medical setting. The goal of “trying out” is to foster relational and interaction component to use that could be continued in a home setting with parent-child interaction. This study purposed to research relaxation apps targeted to pediatric age groups to create a reference guide for families. When families are utilizing technology with children, the format of technology would ideally be not just individual gaming activities but purposeful, relational relaxation using technology tools [24]. 

The apps which most compelled our study team were those with creative, relational use of calming technology for children include Kindoma [9,18], which allows parents to actively color/draw with their child even from a distance, or apps which allow a parent to read a bedtime story to a child from a distance. Calm [13] and Mindshift [17] apps allowed for a speaker version which would allow a parent and child to breathe together for a partnered relaxation approach. Fluid allows more than one finger or hand to pull colors on the screen at one time, allowing a parent and child to co-design calming patterns. 

Although the app search was focused on pediatric age ranges, the calming kinetics of nature imagery, soothing music, and guided meditations have potential to appeal to both pediatric patients and their parental caregivers. Further research will explore the effectiveness of these apps in clinical practice. Next steps for engagement include pilot studies grounded in participatory approach to measure not only the self-reported experience of pediatric palliative care patients and their family members through qualitative inquiry and patient reported outcomes but also meaningful concurrent biometric outcomes such as measured physiologic changes with app use. Most exciting would be the eventual development of a pediatric palliative care app for calming, relaxation, and mindfulness designed with children and families receiving palliative care based on a combined culmination of favorite app features and patient-specific feedback on app quality.

## 5. Conclusions

The provision of a mobile apps resource summary has the potential to foster pediatric palliative care providers’ knowledge of app functionality and applicability as part of ongoing patient care. 

## Figures and Tables

**Table 1:** Role of technology in a pediatric medical setting.

App Role	Relevant Clinical Scenario	Examples of App Technique
Relaxation—Actively fosters entering a state of calm; focusing mind; releasing tension	8-year-old hospitalized oncology patient struggling with insomnia limited to hospital setting 12-year-old female with sickle cell disease reporting increased “all over” body pain during month of parents’ divorce	Mindfulness Yoga/body movement Meditation, hypnosis, or visualization Body scan
Distraction—Passively offers a diversion or recreation for stress reduction and anxiety alleviation	8-year-old male frightened of needles in lab for blood draw 12-year-old female feeling nervous in busy waiting room while waiting to see doctor for scheduled chemotherapy	Games Soothing images Calming audio

**Table 2:** App listing and characteristics.

App Name	Description	Language Available	MARS Engagement	MARS Functionality	MARS Aesthetics	MARS Information	Suggested Age Group	App Approach
Breathe, Think, Do with Sesame [10]	Teaches breathing techniques while offering fun interactive games.	English/Spanish	High 4.6	High 4.7	High 4.7	High 4	Infant, Preschool	Game simulation for breathing exercises
Breathe2Relax [11]	Animated video with demonstration of breathing technique. Customizable breathing app.	English	High 3.8	High 4.8	High 4	High 4.6	Elementary and above	Breathing exercises
Smiling Mind [12]	Guided meditations separated by age group.	English	Medium 3	High 4	High 3.7	High 3.4	Elementary and above	Meditation
Calm [13]	App for mindfulness, meditation, breathing, and improved sleep.	English	High 4.4	High 4.8	High 5	High 3.5	Elementary and above	Meditation and breathing exercises
MyCalmBeat [14]	Breath training to slow breaths to six breaths per minute.	English	High 3.6	High 4.8	High 5	High 4	Elementary and above	Breathing exercises
Nature Sounds [15]	This app offers different relaxing sounds. You can make playlists and save your favorites.	English/Spanish	Medium 3	High 5	High 4.7	High 4.4	Elementary and above	Relaxing audio sounds
Headspace [16]	Teaches a new meditation technique each day.	English	High 4	High 4	High 4	High 3.8	Elementary and above	Meditation
Mindshift [17]	Anxiety specific. Coping with and facing anxiety.	English	High 4.8	High 5	High 4.3	High 4.5	Adolescent and above	Meditation, yoga, and cognitive therapy
Kindoma Drawtime [9]	Video chat for young children and their loved ones to draw together.	English	High 4.2	High 4.3	High 4.7	High 4	All	Real time video chat; relational and creative
Kindoma Storytime [18]	Video chat plus books for young children and their loved ones to read together.	English	High 4.4	High 4.3	High 4.7	High 4	All	Real time video chat; relational
Nature Sounds [19]	Relaxation, using audio sounds, nature images for background.	English	Medium 2.6	High 4	Medium 2.6	Medium 2.3	All	Relaxing audio sounds
Art of Glow [20]	Create kinetic colorful art e.g., fireworks, colors glow.	English	Medium 2.8	High 3.5	Medium 3	Medium 3	All	Relaxing visual images
Nature Sounds Relax and Sleep [21]	Nature sounds to promote relaxation and sleep.	English	Medium 2.8	High 4	Medium 2.3	Medium 2.6	All	Relaxing audio sounds
Relax Melodies [22]	Offers 52 sounds and melodies that can be combined by user preference. Has timer and alarm. Also offers five-day meditation programs.	English/Spanish	High 3.6	High 4.8	High 4.7	High 3.7	All	Relaxing audio sounds
Fluid [8]	Touch screen into liquid surface—makes drops/waves.	English	Medium 3	High 4	Medium 3.3	Medium 2.4	All	Relaxing visual images
Koi Pond Lite [23]	Nature scene, water, fish, flowers—can customize.	English/Spanish	Medium 3	High 4	High 3.6	Medium 2.4	All	Relaxing visual images

MARS: Validated Mobile Application Rating Scale.
